# Supplementary material for: Aquaculture facility-specific microbiota shape the zebrafish gut microbiome
Source: bioRxiv. 2025 Sep 4:2025.09.04.674294. Preprint. [Version 1] doi: 10.1101/2025.09.04.674294 (PMC12424813; doi:10.1101/2025.09.04.674294)
Supplement: Supplement 2 [file media-2.pdf]

Supplementary Table 2: Top 10 most significant ASVs contributing to water microbiome dissimilarity between facility comparisons (SIMPER analysis, $p < 0.05$)

Facility Comparison	Genus	Average Contribution (%)	p-value	Rank
**Ore1 vs Ore2**				
**Ore1 vs Ore2**	Cetobacterium	13.78	0.011	1
**Ore1 vs Ore2**	Vibrio	4.85	0.001	4
**Ore1 vs Ore2**	Plesiomonas	2.58	0.001	8
**Ore1 vs Ore2**	Chitinibacter	1.33	0.001	10
**Ore1 vs Ore2**	Chitinimonas	0.97	0.001	11
**Ore1 vs Ore2**	Crenobacter	0.58	0.001	18
**Ore1 vs Ore2**	Epulopiscium	0.55	0.001	19
**Ore1 vs Ore2**	Haliangium	0.44	0.001	21
**Ore1 vs Ore2**	Rhizorhapis	0.34	0.001	24
**Ore1 vs Ore2**	Romboutsia	0.32	0.001	26
**Ore1 vs Nor1**				
**Ore1 vs Nor1**	Pseudomonas	17.82	0.001	1
**Ore1 vs Nor1**	Stenotrophomonas	2.28	0.026	10
**Ore1 vs Nor1**	Massilia	1.79	0.001	11
**Ore1 vs Nor1**	Variovorax	0.99	0.001	16
**Ore1 vs Nor1**	Nubsella	0.89	0.010	17
**Ore1 vs Nor1**	Deinococcus	0.65	0.001	21
**Ore1 vs Nor1**	Ottowia	0.62	0.001	22
**Ore1 vs Nor1**	Salinirepens	0.61	0.040	23
**Ore1 vs Nor1**	Alkanibacter	0.51	0.001	26
**Ore1 vs Nor1**	Sphingobacterium	0.45	0.001	27
**Ore1 vs Nor2A**				
**Ore1 vs Nor2A**	Aeromonas	6.00	0.027	3
**Ore1 vs Nor2A**	Acidovorax	5.72	0.001	4
**Ore1 vs Nor2A**	Nevskia	5.04	0.006	5
**Ore1 vs Nor2A**	Limnobacter	4.28	0.001	6
**Ore1 vs Nor2A**	Luteimonas	3.10	0.001	12
**Ore1 vs Nor2A**	Stenotrophomonas	2.29	0.033	15
**Ore1 vs Nor2A**	Pelomonas	2.13	0.004	16
**Ore1 vs Nor2A**	Brevundimonas	2.12	0.001	17
**Ore1 vs Nor2A**	Rhodoferax	1.21	0.001	21
**Ore1 vs Nor2A**	Methyloversatilis	1.19	0.003	22
**Ore1 vs Nor2B**				

Facility Comparison	Genus	Average Contribution (%)	p-value	Rank
**Ore1 vs Nor2B**	Rheinheimera	16.91	0.001	1
**Ore1 vs Nor2B**	Acinetobacter	9.13	0.007	4
**Ore1 vs Nor2B**	Fluviicola	5.73	0.001	5
**Ore1 vs Nor2B**	Polynucleobacter	1.38	0.001	12
**Ore1 vs Nor2B**	Sediminibacterium	1.26	0.001	13
**Ore1 vs Nor2B**	hgcI clade	1.15	0.001	14
**Ore1 vs Nor2B**	Candidatus Nitrosotenuis	0.97	0.001	15
**Ore1 vs Nor2B**	Bdellovibrio	0.80	0.001	19
**Ore1 vs Nor2B**	Candidatus Omnitrophus	0.33	0.002	28
**Ore1 vs Nor2B**	Cellvibrio	0.24	0.001	34
**Ore2 vs Nor1**				
**Ore2 vs Nor1**	Cetobacterium	18.11	0.001	1
**Ore2 vs Nor1**	Pseudomonas	18.10	0.001	2
**Ore2 vs Nor1**	Vibrio	4.52	0.007	3
**Ore2 vs Nor1**	Plesiomonas	2.73	0.003	5
**Ore2 vs Nor1**	Stenotrophomonas	2.29	0.033	8
**Ore2 vs Nor1**	Massilia	1.80	0.001	9
**Ore2 vs Nor1**	Variovorax	1.00	0.001	15
**Ore2 vs Nor1**	Nubsella	0.89	0.013	18
**Ore2 vs Nor1**	Deinococcus	0.68	0.001	19
**Ore2 vs Nor1**	Ottowia	0.64	0.001	21
**Ore2 vs Nor2A**				
**Ore2 vs Nor2A**	Cetobacterium	20.33	0.001	1
**Ore2 vs Nor2A**	Acidovorax	5.71	0.001	2
**Ore2 vs Nor2A**	Nevskia	5.06	0.010	3
**Ore2 vs Nor2A**	Vibrio	4.70	0.007	4
**Ore2 vs Nor2A**	Limnobacter	4.57	0.001	5
**Ore2 vs Nor2A**	Plesiomonas	3.24	0.001	9
**Ore2 vs Nor2A**	Luteimonas	3.15	0.001	11
**Ore2 vs Nor2A**	Stenotrophomonas	2.29	0.048	13
**Ore2 vs Nor2A**	Brevundimonas	2.16	0.001	14
**Ore2 vs Nor2A**	Pelomonas	1.86	0.021	15

Facility Comparison	Genus	Average Contribution (%)	p-value	Rank
**Ore2 vs Nor2B**				
**Ore2 vs Nor2B**	Cetobacterium	20.21	0.003	1
**Ore2 vs Nor2B**	Rheinheimera	17.06	0.001	2
**Ore2 vs Nor2B**	Acinetobacter	8.91	0.006	3
**Ore2 vs Nor2B**	Fluviicola	5.72	0.001	4
**Ore2 vs Nor2B**	Vibrio	5.20	0.034	5
**Ore2 vs Nor2B**	Plesiomonas	3.23	0.014	6
**Ore2 vs Nor2B**	Polynucleobacter	1.39	0.001	12
**Ore2 vs Nor2B**	Sediminibacterium	1.29	0.001	14
**Ore2 vs Nor2B**	hgcI clade	1.15	0.002	15
**Ore2 vs Nor2B**	Candidatus Nitrosotenuis	0.96	0.002	17
**Nor2A vs Nor1**				
**Nor2A vs Nor1**	Pseudomonas	14.60	0.001	1
**Nor2A vs Nor1**	Acidovorax	5.38	0.001	2
**Nor2A vs Nor1**	Nevskia	4.91	0.019	3
**Nor2A vs Nor1**	Delftia	4.85	0.002	4
**Nor2A vs Nor1**	Limnobacter	4.31	0.001	5
**Nor2A vs Nor1**	Luteimonas	2.82	0.001	8
**Nor2A vs Nor1**	Stenotrophomonas	2.79	0.014	9
**Nor2A vs Nor1**	Brevundimonas	2.02	0.001	11
**Nor2A vs Nor1**	Pelomonas	1.86	0.035	13
**Nor2A vs Nor1**	Massilia	1.75	0.001	14
**Nor2B vs Nor1**				
**Nor2B vs Nor1**	Pseudomonas	17.49	0.001	1
**Nor2B vs Nor1**	Rheinheimera	16.29	0.001	2
**Nor2B vs Nor1**	Acinetobacter	7.38	0.010	3
**Nor2B vs Nor1**	Fluviicola	5.52	0.001	4
**Nor2B vs Nor1**	Massilia	1.75	0.013	11

Facility Comparison	Genus	Average Contribution (%)	p-value	Rank
**Nor2B vs Nor1**	Polynucleobacter	1.33	0.017	14
**Nor2B vs Nor1**	Sediminibacterium	1.18	0.001	15
**Nor2B vs Nor1**	hgcI clade	1.10	0.005	18
**Nor2B vs Nor1**	Variovorax	0.98	0.048	19
**Nor2B vs Nor1**	Candidatus Nitrosotenuis	0.94	0.002	20
**Nor2A vs Nor2B**				
**Nor2A vs Nor2B**	Rheinheimera	13.42	0.001	1
**Nor2A vs Nor2B**	Acinetobacter	8.32	0.003	2
**Nor2A vs Nor2B**	Fluviicola	5.61	0.001	3
**Nor2A vs Nor2B**	Luteimonas	3.06	0.006	8
**Nor2A vs Nor2B**	Brevundimonas	2.04	0.050	13
**Nor2A vs Nor2B**	Polynucleobacter	1.35	0.017	17
**Nor2A vs Nor2B**	hgcI clade	1.12	0.001	20
**Nor2A vs Nor2B**	Sediminibacterium	1.07	0.002	21
**Nor2A vs Nor2B**	Candidatus Nitrosotenuis	0.96	0.001	22
**Nor2A vs Nor2B**	Bdellovibrio	0.83	0.001	24
