# Supplementary material for: Aquaculture facility-specific microbiota shape the zebrafish gut microbiome
Source: bioRxiv. 2025 Sep 4:2025.09.04.674294. Preprint. [Version 1] doi: 10.1101/2025.09.04.674294 (PMC12424813; doi:10.1101/2025.09.04.674294)
Supplement: Supplement 3 [file media-3.pdf]

Supplementary Table 3: Top 10 most significant ASVs contributing to fish gut microbiome dissimilarity between facility comparisons (SIMPER analysis, p < 0.05)

Facility Comparison	Genus	Average Contribution (%)	p-value	Rank
**Ore1 vs Ore2**				
**Ore1 vs Ore2**	Streptococcus	5.94	0.001	3
**Ore1 vs Ore2**	Enterococcus	5.53	0.001	4
**Ore1 vs Ore2**	Vagococcus	0.74	0.008	12
**Ore1 vs Ore2**	Epulopiscium	0.33	0.016	19
**Ore1 vs Ore2**	Gemmobacter	0.10	0.003	41
**Ore1 vs Ore2**	Clostridium sensu stricto 13	0.09	0.008	42
**Ore1 vs Ore2**	Isosphaera	0.09	0.012	43
**Ore1 vs Ore2**	Rhodopirellula	0.04	0.030	53
**Ore1 vs Ore2**	Paeniglutamicibacter	0.02	0.003	78
**Ore1 vs Ore2**	Gaiella	0.01	0.002	101
**Ore1 vs Nor1**				
**Ore1 vs Nor1**	Cetobacterium	26.59	0.001	1
**Ore1 vs Nor1**	Pseudomonas	9.14	0.001	3
**Ore1 vs Nor1**	Lactococcus	3.47	0.001	5
**Ore1 vs Nor1**	Streptococcus	2.45	0.009	7
**Ore1 vs Nor1**	Achromobacter	2.38	0.001	8
**Ore1 vs Nor1**	Pediococcus	2.25	0.001	9
**Ore1 vs Nor1**	Staphylococcus	1.97	0.003	10
**Ore1 vs Nor1**	Leuconostoc	1.72	0.001	13
**Ore1 vs Nor1**	Latilactobacillus	1.23	0.008	14
**Ore1 vs Nor1**	Nocardia	1.10	0.008	15
**Ore1 vs Nor2A**				
**Ore1 vs Nor2A**	Vibrio	23.29	0.001	1
**Ore1 vs Nor2A**	Cetobacterium	22.95	0.001	2
**Ore1 vs Nor2A**	Bordetella	1.25	0.041	8
**Ore1 vs Nor2A**	Pseudonocardia	0.48	0.035	18
**Ore1 vs Nor2A**	Paucilactobacillus	0.13	0.048	38
**Ore1 vs Nor2A**	Gallicola	0.08	0.043	46
**Ore1 vs Nor2A**	Pedomicrobium	0.05	0.020	53
**Ore1 vs Nor2A**	Micrococcus	0.00	0.005	164
**Ore1 vs Nor2B**				
**Ore1 vs Nor2B**	Aeromonas	27.07	0.001	1
**Ore1 vs Nor2B**	Cetobacterium	26.43	0.001	2

Facility Comparison	Genus	Average Contribution (%)	p-value	Rank
**Ore1 vs Nor2B**	Bacillus	2.54	0.018	5
**Ore1 vs Nor2B**	Crenobacter	0.64	0.001	14
**Ore1 vs Nor2B**	Chelativorans	0.60	0.001	15
**Ore1 vs Nor2B**	Marmoricola	0.37	0.001	20
**Ore1 vs Nor2B**	Pir4 lineage	0.30	0.029	24
**Ore1 vs Nor2B**	Chitinilyticum	0.24	0.001	27
**Ore1 vs Nor2B**	Hyphomicrobium	0.21	0.045	30
**Ore1 vs Nor2B**	Flavobacterium	0.19	0.001	33
**Ore2 vs Nor1**				
**Ore2 vs Nor1**	Cetobacterium	22.38	0.042	1
**Ore2 vs Nor1**	Streptococcus	5.90	0.003	5
**Ore2 vs Nor1**	Enterococcus	5.25	0.001	6
**Ore2 vs Nor1**	Vagococcus	0.87	0.001	16
**Ore2 vs Nor1**	Epulopiscium	0.27	0.050	27
**Ore2 vs Nor1**	Gemmobacter	0.09	0.006	41
**Ore2 vs Nor1**	Clostridium sensu stricto 13	0.09	0.008	43
**Ore2 vs Nor1**	Isosphaera	0.08	0.026	45
**Ore2 vs Nor1**	Paeniglutamicibacter	0.02	0.040	83
**Ore2 vs Nor1**	Clostridium sensu stricto 18	0.01	0.040	101
**Ore2 vs Nor2A**				
**Ore2 vs Nor2A**	Vibrio	20.24	0.018	1
**Ore2 vs Nor2A**	Streptococcus	6.20	0.002	4
**Ore2 vs Nor2A**	Enterococcus	5.60	0.001	5
**Ore2 vs Nor2A**	Vagococcus	0.86	0.002	14
**Ore2 vs Nor2A**	Gemmobacter	0.09	0.013	23
**Ore2 vs Nor2A**	Clostridium sensu stricto 13	0.09	0.013	24
**Ore2 vs Nor2A**	Isosphaera	0.08	0.026	25
**Ore2 vs Nor2A**	Paeniglutamicibacter	0.02	0.041	47
**Ore2 vs Nor2A**	Clostridium sensu stricto 18	0.01	0.041	60
**Ore2 vs Nor2A**	Desulfotomaculum	0.01	0.041	64
**Ore2 vs Nor2B**				

Facility Comparison	Genus	Average Contribution (%)	p-value	Rank
**Ore2 vs Nor2B**	Aeromonas	27.87	0.001	1
**Ore2 vs Nor2B**	Streptococcus	6.61	0.001	3
**Ore2 vs Nor2B**	Enterococcus	5.97	0.001	5
**Ore2 vs Nor2B**	Vagococcus	0.92	0.002	10
**Ore2 vs Nor2B**	Crenobacter	0.60	0.036	11
**Ore2 vs Nor2B**	Chelativorans	0.59	0.009	12
**Ore2 vs Nor2B**	Marmoricola	0.36	0.008	15
**Ore2 vs Nor2B**	Epulopiscium	0.29	0.048	17
**Ore2 vs Nor2B**	Chitinilyticum	0.24	0.029	18
**Ore2 vs Nor2B**	Flavobacterium	0.19	0.042	19
**Nor2A vs Nor1**				
**Nor2A vs Nor1**	Vibrio	23.79	0.001	1
**Nor2A vs Nor1**	Pseudomonas	9.80	0.001	3
**Nor2A vs Nor1**	Lactococcus	3.22	0.022	5
**Nor2A vs Nor1**	Stenotrophomonas	2.75	0.045	6
**Nor2A vs Nor1**	Delftia	2.73	0.018	7
**Nor2A vs Nor1**	Pediococcus	2.18	0.005	10
**Nor2A vs Nor1**	Leuconostoc	1.67	0.006	13
**Nor2A vs Nor1**	Bordetella	1.26	0.033	14
**Nor2A vs Nor1**	Limosilactobacillus	0.27	0.005	28
**Nor2A vs Nor1**	Pedomicrobium	0.05	0.007	45
**Nor2B vs Nor1**				
**Nor2B vs Nor1**	Aeromonas	20.17	0.001	1
**Nor2B vs Nor1**	Pseudomonas	9.41	0.014	2
**Nor2B vs Nor1**	Lactococcus	3.43	0.040	4
**Nor2B vs Nor1**	Bacillus	2.48	0.030	7
**Nor2B vs Nor1**	Pediococcus	2.32	0.014	10
**Nor2B vs Nor1**	Leuconostoc	1.78	0.017	14

Facility Comparison	Genus	Average Contribution (%)	p-value	Rank
**Nor2B vs Nor1**	Candidatus Nitrocosmicus	1.50	0.016	15
**Nor2B vs Nor1**	Mycobacterium	0.78	0.011	16
**Nor2B vs Nor1**	Crenobacter	0.67	0.001	17
**Nor2B vs Nor1**	Chelativorans	0.59	0.001	19
**Nor2A vs Nor2B**				
**Nor2A vs Nor2B**	Vibrio	23.66	0.001	1
**Nor2A vs Nor2B**	Aeromonas	23.65	0.001	2
**Nor2A vs Nor2B**	Bacillus	3.34	0.002	5
**Nor2A vs Nor2B**	Crenobacter	0.62	0.001	12
**Nor2A vs Nor2B**	Chelativorans	0.58	0.001	13
**Nor2A vs Nor2B**	Marmoricola	0.36	0.001	18
**Nor2A vs Nor2B**	Chitinilyticum	0.23	0.001	21
**Nor2A vs Nor2B**	Flavobacterium	0.18	0.001	22
**Nor2A vs Nor2B**	Pirellula	0.17	0.001	23
**Nor2A vs Nor2B**	Nannocystis	0.14	0.002	26
